# Comparative Proteomic Analysis of the Molecular Responses of Mouse Macrophages to Titanium Dioxide and Copper Oxide Nanoparticles Unravels Some Toxic Mechanisms for Copper Oxide Nanoparticles in Macrophages

**DOI:** 10.1371/journal.pone.0124496

**Published:** 2015-04-22

**Authors:** Sarah Triboulet, Catherine Aude-Garcia, Lucie Armand, Véronique Collin-Faure, Mireille Chevallet, Hélène Diemer, Adèle Gerdil, Fabienne Proamer, Jean-Marc Strub, Aurélie Habert, Nathalie Herlin, Alain Van Dorsselaer, Marie Carrière, Thierry Rabilloud

**Affiliations:** 1 Laboratory of Chemistry and Biology of Metals, Univ. Grenoble Alpes, Grenoble, France; 2 Laboratory of Chemistry and Biology of Metals, CEA/ iRTSV, Grenoble, France; 3 Laboratory of Chemistry and Biology of Metals, CNRS UMR5249, Grenoble, France; 4 Service de Chimie Inorganique et Biologique, Univ. Grenoble Alpes & CEA, Grenoble, France; 5 Laboratoire de Spectrométrie de Masse BioOrganique (LSMBO), Université de Strasbourg & CNRS UMR 7178, Institut Pluridisciplinaire Hubert Curien, Strasbourg, France; 6 Laboratoire Francis Perrin (Unité de recherche Associée 2453), Commissariat à l’Energie Atomique, CEA-Saclay 91191 Gif/Yvette, France; 7 Etablissement Français du Sang-Alsace, Unité MIxte de recherche S949 Institut National de la Santé Et de la Recherche Médicale (INSERM)-Université de Strasbourg, Strasbourg, France

## Abstract

Titanium dioxide and copper oxide nanoparticles are more and more widely used because of their catalytic properties, of their light absorbing properties (titanium dioxide) or of their biocidal properties (copper oxide), increasing the risk of adverse health effects. In this frame, the responses of mouse macrophages were studied. Both proteomic and targeted analyses were performed to investigate several parameters, such as phagocytic capacity, cytokine release, copper release, and response at sub toxic doses. Besides titanium dioxide and copper oxide nanoparticles, copper ions were used as controls. We also showed that the overall copper release in the cell does not explain per se the toxicity observed with copper oxide nanoparticles. In addition, both copper ion and copper oxide nanoparticles, but not titanium oxide, induced DNA strands breaks in macrophages. As to functional responses, the phagocytic capacity was not hampered by any of the treatments at non-toxic doses, while copper ion decreased the lipopolysaccharide-induced cytokine and nitric oxide productions. The proteomic analyses highlighted very few changes induced by titanium dioxide nanoparticles, but an induction of heme oxygenase, an increase of glutathione synthesis and a decrease of tetrahydrobiopterin in response to copper oxide nanoparticles. Subsequent targeted analyses demonstrated that the increase in glutathione biosynthesis and the induction of heme oxygenase (e.g. by lovastatin/monacolin K) are critical for macrophages to survive a copper challenge, and that the intermediates of the catecholamine pathway induce a strong cross toxicity with copper oxide nanoparticles and copper ions.

## Introduction

Because of their increasing use in various products, nanoparticles have been very intensively studied on a toxicological point of view, with a special emphasis on pulmonary toxicity. The results described to date have been widely divergent, even for a given nanoparticle. For example, some results showed a strong pulmonary toxicity for titanium dioxide nanoparticles [1, 2] while others concluded to a low toxicity [3–5]. This major discrepancy has been linked to the mode of administration *in vivo* [6, 7], as well as to widely divergent doses used in the various studies.

Moreover, these in vivo studies usually do not provide molecular mechanisms as to the responses of cells to the nanoparticles.

As to in vitro studies, one of the the key cell types of interest is macrophages, which play a major role in the clearance of particles in the lung, including titanium dioxide nanoparticles [8], but also in several pulmonary diseases, as documented for example in the case of asbestos [9]. Other dysfunctions of the innate immune system can lead to deregulation of the immune responses and to severe adverse effects, e.g. a higher incidence of tumours [10].

It is therefore not surprising that immunotoxicology of nanoparticles is a developing field (e.g. in [11]), and several studies have been devoted to the response of macrophages to nanoparticles. Within them, several have studied titanium dioxide [12–20], here again with quite divergent conclusions regarding the toxicity of titanium dioxide nanoparticles, at least in this cell type.

Among the in vitro studies of the cellular responses to nanoparticles, very few use the analytical power of omics to go deeper into the cellular responses to nanoparticles. A few exceptions exist, however. Proteomic analyses have been conducted on carbon-based nanoparticles [21], on titanium dioxide [13, 22, 23] and on airborne particulate matter [24]. However, it is surprising to note that most of these proteomic analyses have been performed on BEAS-2B bronchial epithelial cells [13, 22, 24, 25] or on complete lung tissue [23], with only one study carried out on carbon nanotubes and on the U937 monocyte model [21]. In fact no proteomic study has been carried out on the effect of titanium dioxide nanoparticles on macrophages, and only a transcriptomic work has been recently described [19], concluding to very limited effects of titanium dioxide nanoparticles on immune cells.

Comparatively to titanium dioxide, copper oxide nanoparticles are much less used in industry and much less studied regarding their interaction with living cells and organisms. However, copper oxide nanoparticles are much more toxic than titanium dioxide nanoparticles [3, 26, 27]. Their toxicity is attributed to the release of copper ion inside cells [28, 29]. However, the toxicity of copper oxide does not superpose to the one of copper sulfate [30], copper oxide being more toxic than copper salts.

Both transcriptomic and proteomic analyses have been performed to study the cellular responses to copper oxide [31–33], and have highlighted various responding pathways. However, as these omics techniques are very context-sensitive, a comparison between different nanoparticles would be of interest to highlight the specific pathways for each nanoparticle, as well as the common pathways induced, for example, by the phagocytosis event per se. This comparative analysis has been carried out on titanium dioxide vs. zinc oxide [19], but is lacking so far for copper oxide. This is why we This is why we carried out this comparison for copper oxide vs titanium dioxide, on the macrophage cell line J774, using a proteomic approach.

## Materials and Methods

### Nanoparticles

Titanium dioxide nanoparticles were purchased directly as a suspension in water (Sigma Aldrich ref # 700347). They were diluted to 5% w/v (final concentration) in a water solution containing 2% w/v dextran (final concentration), and the particles were coated for 1 hour under constant agitation. The solution was then sterilized by incubation at 90°C for 2 hours. This step was required to prevent any contamination of the dispersion with microorganisms, as many bacterial or fungal products such as lipopolysaccharide induce major responses in macrophages.

The copper oxide nanoparticles (<50 nm) were purchased from Sigma-Aldrich (ref # 544868). They were dispersed in water as a 5.5% w/v suspension by sonication for 60 minutes in a cup-horn instrument (BioBlock Scientific, France), under a 5°C thermostated water circulation. 0.1 volume of a 10% w/v PVP40 solution (as dextran was unable to keep copper oxide nanoparticles dispersed in culture media) was added under sterile conditions, and the particles were coated for 1 hour under constant agitation.

The actual size of the particles was determined after dilution in water or in complete culture medium by dynamic light scattering, using a Wyatt Dynapro Nanostar instrument. A Malvern HS 3000 instrument was also used to determine the zeta potential. The crystalline phases were identified using a powder diffractometer from Bruker and the Match software (crystal impact). Samples morphology was observed by SEM (Scanning Electron Microscopy) and TEM (Transmission Electron Microscopy) after dispersion of the nanoparticles in water using an ultrasonic bath and deposition of a droplet on 200 mesh carbon lacey grids.

### Cell culture

#### Cell lines

The mouse macrophage cell line J774A1 was purchased from the European Cell Culture Collection (Salisbury, UK). The cells were cultured in RPMI 1640 medium + 10% fetal bovine serum. Cells were seeded every two days at 200,000 cells/ml and harvested at 1,000,000 cells per ml. For treatment with nanoparticles, the following scheme was used: cells were first seeded at 500,000 cells/ml in T175 flasks (50 ml per flask). They were exposed to nanoparticles on the following day and harvested after a further 24 hours in culture. Cell viability was measured by trypan blue exclusion. For treatment with inhibitors, cells were pretreated for 6 hours with either 10μM buthionine sulfoximine or 2μM lovastatin,100μM DOPA, 2mM tyrosine or 200μM 6-hydroxytryptophan. Copper oxide nanoparticles or copper chloride or titanium dioxide nanoparticles were then added for a further 18 hours before collection of the cells and viability measurements.

All experiments were carried out at least in triplicate on independent cultures.

#### Transmission Electron Microscopy on cells

Transmisson electron microscopy was carried out as previously described [32] on cells fixed with glutaraldehyde, included in Epon and post stained with lead citrate and uranyl acetate. Further details can be found in the S1 Methods.

#### Nanoparticles dissolution in cells

For the studies on cell extracts, the cells were treated with copper oxide nanoparticles (or copper acetate) for 24 hours prior to harvest. The cells were then recovered by scraping, collected by centrifugation, washed three times with PBS, and the volume of the cell pellet was determined. A cell lysis solution (Hepes 20mM pH 7.5, MgCl2 2mM, KCl 50 mM, tetradecyldimethylammonio propane sulfonate (SB 3–14) 0.15% w/v) was added at a ratio of 5 volumes of solution per volume of cell pellet, and the cells were left to lyse on ice for 20 minutes. The suspension was then centrifuged at 1000g for 5 minutes, the supernatant collected, and recentrifuged at 270,000g for 45 minutes to sediment the nanoparticles [31].

The dissolved copper concentration was then determined using a colorimetric assay [34]. Briefly, 1ml of cell extract was first cooled in an ice bath for 30 minutes. TCA was added to a 5% w/v final concentration, and the mixture was left for 1 hour on ice to denature the proteins and release complexed copper ion. The mixture was centrifuged for 10 minutes at 15,000g, the supernatant recovered and its volume measured. A neutralizing solution (2M MES-Na salt) was added (0.25 volume/volume of supernatant), followed by 0.01 volume of BCA solution (2.5 mg/ml BCA in DMSO) and 0.015 volume of sodium ascorbate solution (0.5M in water). The color was left to develop for 10 minutes and the absorbance measured at 360nm. Under these conditions, no interference was observed with magnesium, calcium, zinc or iron. This assay was chosen for its good correlation with AAS even in complex biological media [34], and for the fact that opposite to mineralization strategies, it will be much less sensitive to interference by remaining particles.

#### Phagocytosis activity and cytokine measurements

The phagocytic activity was measured after treatment with particles or copper ions using fluorescent latex beads (1μm diameter, green labelled, catalog number L4655 from Sigma) and flow cytometry, essentially as described in [32, 35]. The cytokines were measured with a cytokine bead array kit (Becton Dickinson) used according to the manufacturer's instructions. Further details can be found in the S1 Methods.

#### Comet assay

The comet assay was performed in its alkaline version, essentially as described in Jugan et al. [36]. Briefly, microscope slides were coated with 1% normal melting point agarose (NMA) and allowed to dry. Around 10,000 cells (75 μL of each cell suspension) were mixed with 0.6% low melting point agarose (LMPA) and deposited over the agarose layer, and the LMPA/cells mix was allowed to solidify on ice. The slides were immersed overnight in cold lysis solution (2.5 M NaCl, 100 mM EDTA, 10 mM Tris, 1%SDS, 10% DMSO, 1% Triton X-100) at 4°C. DNA was then allowed to unwind for 30 min in alkaline electrophoresis solution (300 mM NaOH, 1 mM EDTA, pH > 13). Electrophoresis was performed in a field of 0.7 V/cm and 300 mA current for 30 min. Slides were then neutralized with 0.4 M Tris pH 7.5 and stained with 50 μL of 20 μg/ml ethidium bromide. At least 50 comets per slide were analyzed under a fluorescence microscope (Zeiss) equipped with a 350–390 nm excitation and a 456 nm emission filter at ×20 magnification. Percentage of tail DNA was measured by using Comet IV software (Perceptive Instruments, Suffolk, UK).

### Proteomics

Sample preparation, 2D gel analysis and mass spectrometry were performed essentially as previously described [32] and the detailed procedures can be found in the S1 Methods. Thus, only the specific methods will be described in detail in this section.

#### Sample preparation

The cells were collected by scraping, and then washed three times in PBS. The cells were then washed once in TSE buffer (Tris-HCl 10 mM pH 7.5, sucrose 0.25M, EDTA 1 mM), and the volume of the cell pellet was estimated. The pellet was resuspended in its own volume of TSE buffer. Then 4 volumes (respective to the cell suspension just prepared) of concentrated lysis buffer (urea 8.75M, thiourea 2.5M, CHAPS 5% w/v, TCEP HCl, 6.25mM, spermine base 12.5mM) were added and the solution was let to extract at room temperature for 1 hour. The nucleic acids were then pelleted by ultracentrifugation (270,000 g at room temperature for 1 hour), and the protein concentration in the supernatant was determined by a dye-binding assay [37]. Carrier ampholytes (Pharmalytes pH 3–10) were added to a final concentration of 0.4% (w/v), and the samples were kept frozen at -20°C until use

#### 2D gel electrophoresis

Isoelectric focusing was carried out on home made 160 mm long 4–8 linear pH gradient gels [38], cast according to published procedures [39], and rehydrated overnight with the sample in 7 M urea, 2 M thiourea, 4% CHAPS, 0.4% carrier ampholytes (Pharmalytes 3–10) and 100mM dithiodiethanol [40, 41].

IEF strips were run for 60–70 kVh (see S1 Methods for details), equilibrated for 20 minutes in Tris 125mM, HCl 100mM, SDS 2.5%, glycerol 30% and urea 6 M [42], and transferred on top of the SDS gels.

Ten percent gels (160x200x1.5 mm) were used for the second dimension. The Tris taurine buffer system [43] was used and operated at a ionic strength of 0.1 and a pH of 7.9. Detection was carried out by fast silver staining [44].

#### Image analysis

The gel images were analyzed using the Delta 2D software (v 3.6). Three gels coming from three independent cultures were used for each experimental group. Spots that were never expressed above 100 ppm of the total spots were first filtered out. Then, significantly-varying spots were selected on the basis of their Student T-test p-value between the treated and the control groups. Spots showing a p-value lower than 0.05 were selected.

#### Mass spectrometry

The spots selected for identification were analyzed by NanoLC-MS/MS analysis, performed using a nanoLC-QTOF-MS system and a nanoLC-IT-MS system operated as described previously [32] (details can also be found in the S1 Methods).

The MS/MS data were interpreted using MASCOT 2.4.0 (Matrix Science, London, UK) against UniProtKB/SwissProt (version 2012_08, 537,505 sequences). The search was carried out in all species. A maximum of one trypsin missed cleavage was allowed. Spectra from Qtof were searched with a mass tolerance of 15 ppm for MS and 0.05 Da for MS/MS data and spectra from Ion Trap were searched with a mass tolerance of 0.3 Da in MS and MS/MS modes. Carbamidomethylation of cysteine residues and oxidation of methionine residues were specified as variable modifications. Protein identifications were validated with at least two peptides with Mascot ion score above 20.

## Results

### Nanoparticles characterization

The two commercial nanoparticles were characterized by several methods, and the results are summarized in Table 1 and in S1 Fig. Both nanoparticles were of a spheroidal shape. The primary titanium dioxide nanoparticles were of uniform size (mean 25nm) whereas the copper oxide nanoparticles showed two populations, of ca. 15–20nm for the smaller one and of ca. 70nm for the larger one, with an overall mean diameter of ca. 30 nm. The XRD diagrams conformed to those of nominal copper oxide and of anatase, showing no crystalline impurities. Once coated with the appropriate polymer, the nanoparticles were stable in complete culture medium as aggregates of ca. 150 nm in diameter for titanium dioxide and of ca. 250nm in diameter for copper oxide. Our results of copper oxide nanoparticles were in complete agreement with those previously reported by other groups with the same brand of copper oxide nanoparticles [31, 45].

**Table 1:** Characterization of the two nanoparticles used in this study.

	CuO	TiO2
Shape	spheroid	spheroid
size of primary particles (TEM)	22±5 nm	25±10 nm
	70±27 nm	
zeta potential	+3.3 mV	-11.3 mV
XRD	conform	Anatase
crystaline domain size (XRD)	ca.18 nm	ca.26 nm
average size (diameter) in water (DLS)*	210± 20 nm	80±10 nm
average size (diameter) in water (DLS)†	250 ± 17 nm	130±10 nm
average size (diameter) in culture medium†	270 ± 10 nm	135±5 nm

*: Size for uncoated nanoparticles

†: Size for coated nanoparticles. The sizes are constant across 24 hours in complete culture medium. Both TiO2 and CuO particles aggregate to a size >1μm if diluted in complete culture medium without prior coating.

### Determination of the effective doses

**Fig 1:**
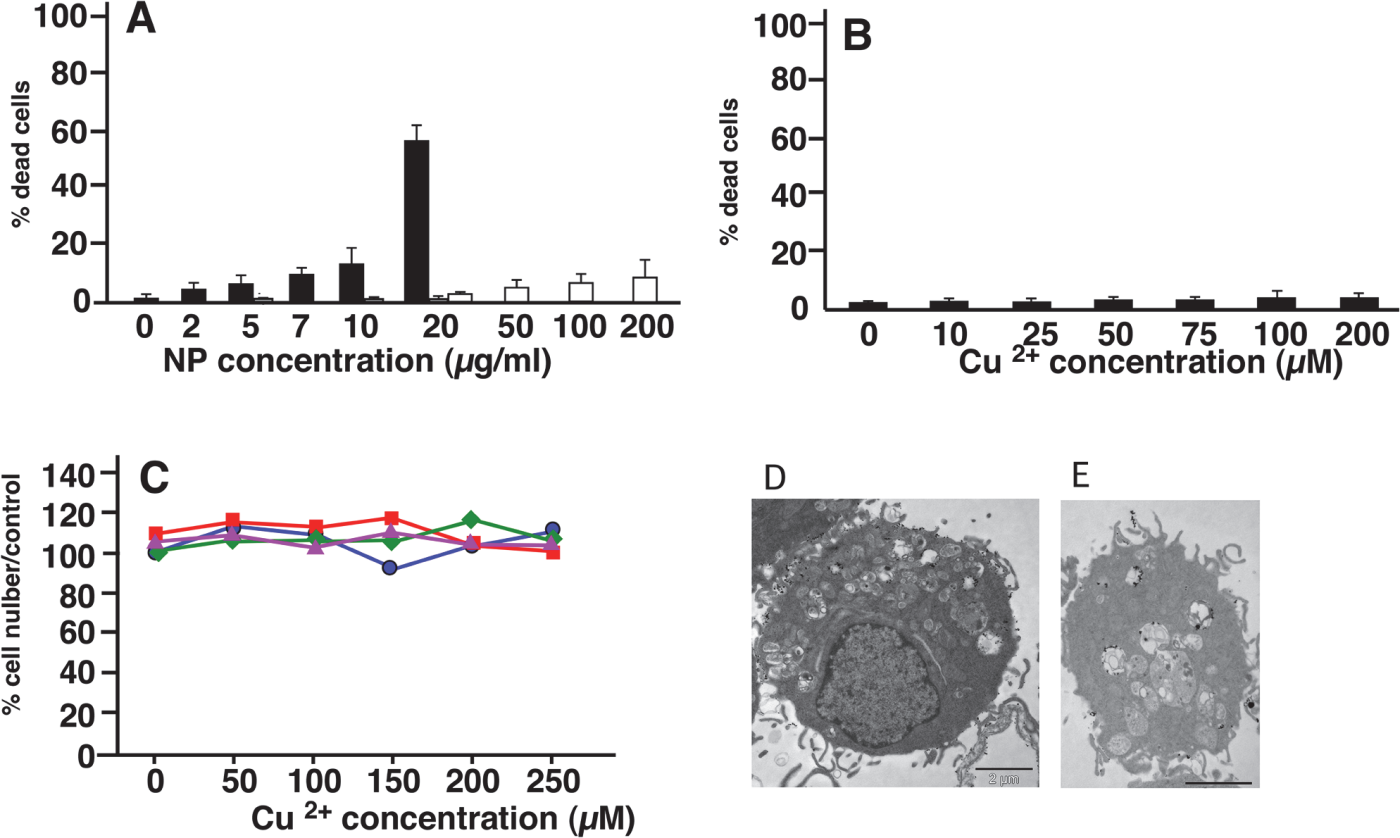
Determination of the cell viability after treatment with copper oxide nanoparticles (A), and copper acetate (B). Experiments were carried out in quadruplicate on independent cultures. Insignificant toxicity was induced by titanium dioxide up to 100μg/ml and by copper oxide microparticles up to 50μg/ml. Grey bars show the absence of toxicity of PVP at the concentrations present in the copper oxide dispersions (respectively1, 2 and 4 μg/ml). In C, surviving cell numbers after a combined treatment by copper acetate alone (blue curve, circles), copper acetate + 25 μg/ml titanium dioxide (red curve, squares), copper acetate + 10μg/ml 250nm latex beads (green curve, diamonds), copper acetate + 25μg/ml 1μm latex beads (purple curve, triangles). None of the observed variation was significant (Mann Whitney U test). In D and E, Transmission electron microscopy of J774 cells exposed to 50μg/ml titanium dioxide nanoparticles for 18 hours (D) or to 10μg/ml copper oxide (E).

For carrying out a proteomics study, we had to determine the dose for which we would obtain the best compromise between viability and biological effect. We therefore decided to use a LD20 if cytotoxic effects were observed, i.e. a dose leading to a cell mortality of 20%, knowing that the mortality of a control culture is around 5%. As shown in Fig 1, the LD20 was obtained at 10μg/ml for CuO nanoparticles (i.e. 125μM) (Fig 1A), and was not reached at 200μM Cu acetate (Fig 1B) or 200μg/ml titanium dioxide nanoparticles (data not shown). These results show a sharp difference between the toxicity of copper oxide nanoparticles and copper ion, in line with what has been observed on HepG2 cells [46], but quite different from the situation observed on other cellular models such as Caco-2 [47] or A549 [48]. Thus, the comparative toxicity of copper ion and copper nanoparticles seems to be cell type-dependent.

As to titanium dioxide, no toxic effect was observed even at 200μg/ml. A dose of 100μg/ml was selected in order to allow cell sorting on the basis of titanium dioxide nanoparticles ingestion. We also checked that both copper oxide nanoparticles and titanium dioxide nanoparticles were efficiently internalized by the cells (Fig 1D and 1E), showing that the different coatings required to disperse the nanoparticles (PVP vs dextran) did not interfere with the internalization process. Moreover, this absence of toxicity of these titanium dioxide nanoparticles under these precise conditions enabled us to use them as a negative control to sort out the generic effects induced by the phagocytosis event from the specific effects generated by copper oxide nanoparticles.

### Determination of copper dissolution in cells

The intracellular uptake of copper oxide and titanium dioxide nanoparticles has been amply demonstrated (e.g. in [32, 49]). As copper oxide nanoparticles are known to dissolve in culture media [29], we decided to investigate how copper oxide nanoparticles dissolve in a cell after internalization. We measured the copper concentration that could be released from a cytosolic extract prepared from cells exposed for 24h to copper ion or CuO nanoparticles. The results, shown in Table 2, demonstrate that both the ion and the nanoparticles induced a comparable accumulation. However, the toxicity did not correlate well with copper ion accumulation. At equal toxicity (LD20), CuO nanoparticles induced a lesser accumulation than the one induced by an ionophore (8-hydroquinoline)-copper ion couple [50]. Conversely, a higher intracellular copper accumulation can be induced by treating the cells with a non toxic concentration of copper ion (150μM) than by treating the cells with a lower concentration of CuO nanoparticles (125μM Cu), which begins to induce toxicity.

**Table 2:** Copper dissolution in cells.

Input	copper
	concentration
	(pmole Cu/mg protein)
Control	256±2
CuONP (10μg/ml)	1370±50
Cu acetate 100μM	960±120
Cu acetate 125μM	1140±190
Cu acetate 150μM	1440±50
Cu acetate 2μM	1800±200
& 8OH-Q 5μM	

J774 cells were treated for 24 hours with the indicated copper concentrations. Cells were then harvested, lysed and cleared by centrifugation, before measurement of the dissolved copper

8OHQ: 8-hydroxyquinoline (copper ionophore)

Altogether, these results go against the "trojan horse mechanism" model, in which the nanoparticulate copper is ingested via phagocytosis or endocytosis and overflows the cell upon dissolution in the acidic cellular compartments (e.g. lysosomes) [51, 52]. Our results are more in line with other published work that does not show a strong correlation between a possible differential copper uptake and toxicity [48].

Thus, an alternative hypothesis to explain the selective toxicity of CuO nanoparticles could be that phagocytosis of particles drives the cells into a state where they become more vulnerable to copper ion. To test this hypothesis, we exposed the cells to various concentrations of copper ion (up to 250μM) and at the same time to non toxic doses of particles, either titanium dioxide nanoparticles (50μg/ml) or to latex nanoparticles, classically used to induce phagocytosis in macrophages (10μg/ml). Both 0.24 and 1μm latex beads were tested. None of these treatments induced any significantly different cell death compared to control cells (Fig 1C). This ruled out the hypothesis of a toxicity caused by a special physiological state induced by the phagocytosis phenomenon. Altogether, these results favor a mechanism in which the way copper is delivered into the cell and its subcellular localization play an important role in the toxicity induced by copper oxide nanoparticles.

### Characterization of the nanoparticles-induced DNA strand breaks

**Fig 2:**
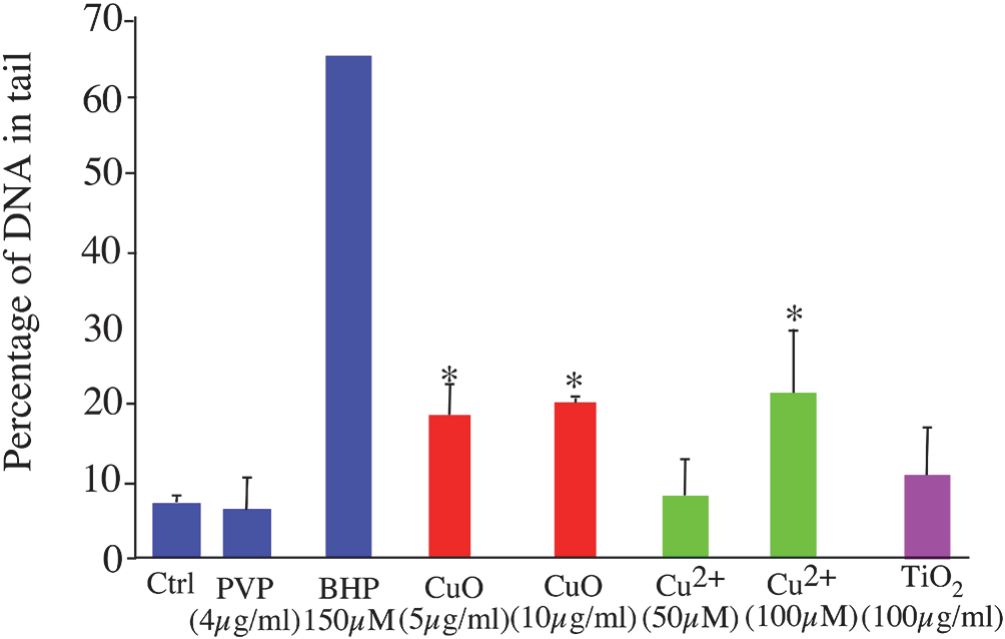
Assessment of DNA damage. Damages to DNA on J774 cells after treatment with copper oxide nanoparticles, copper ion and titanium dioxide nanoparticles were evaluated using the alkaline version of the comet assay, taking into account both single and double strand breaks. The results are expressed in percentage of DNA in the tail. Butylhydroperoxide (BHP) was used as a positive control. Measurements were carried out in triplicate (except for BHP where n = 1). Statistical confidence (Student T-test) is indicated as follows: *: p ≤ 0.05; ** p ≤ 0.01; *** p≤ 0.001

Genotoxicity has been described on several cellular models, both for titanium dioxide [36, 53–56] and for copper oxide nanoparticles [27, 57]. We decided to test comparatively the DNA strand breaks induced by titanium dioxide and copper oxide nanoparticles on macrophages, as this cell type is strongly radioresistant [58], and thus much more resistant to DNA damaging agents. To this effect, we used the comet assay, in its alkaline version, which measures the level of single and double strand breaks as well as alkali-labile sites such as abasic sites [59]. The results (Fig 2) show that copper oxide nanoparticles and copper ion induced DNA strand breaks in macrophages, while titanium dioxide and PVP (polyvinyl pyrrolidone, the coating agent for the copper oxide nanoparticles) were innocuous on this cell type. This strong effect of copper is to be related with the redox potential of the cuprous/cupric couple, which allows redox cycling in Fenton type reactions, producing DNA damaging reactive oxygen species.

### Proteomic analyses

**Fig 3:**
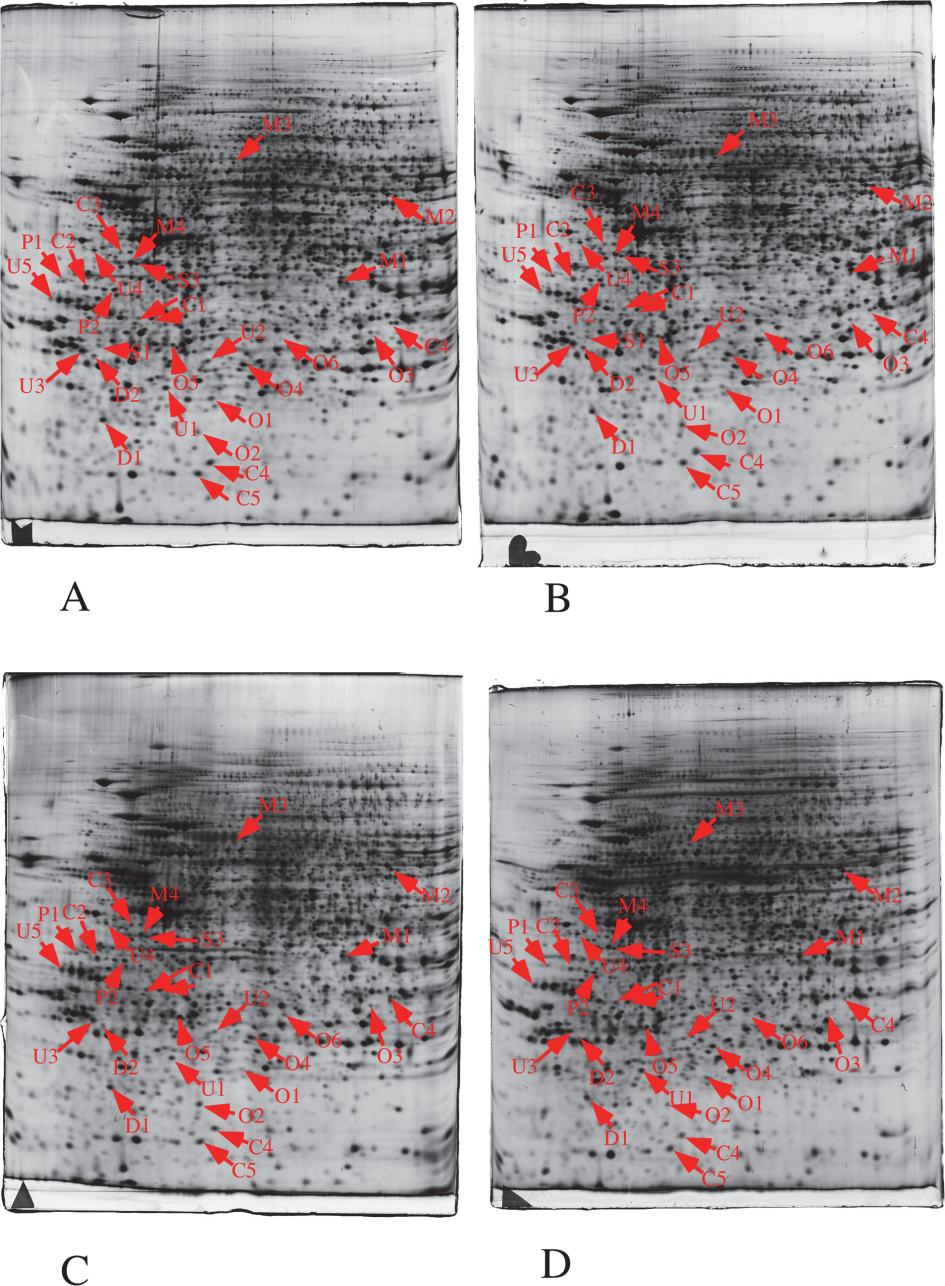
Proteomic analysis of total cell extracts by 2D electrophoresis. Total cell extracts of J774 cells were separated by two-dimensional gel electrophoresis. The first dimensions covered a 4–8 pH range and the second dimension a 15–200 kDa range. Total cellular proteins (150 μg) were loaded on the first dimension gel. A: gel obtained from control cells. B: gel obtained from cells treated with titanium oxide (100μg/ml, 24 hours). C: gel obtained from cells treated with copper oxide (10μg/ml, 24 hours). D: gel obtained from cells treated with copper oxide (125μM, 24 hours). The arrows point to spots that show reproducible and statistically significant changes between the control and NP-treated cells (p≤ 0.05). Spot numbering according to Table 3.

In these experiments, we performed a quantitative analysis of the proteome on whole cell extracts prepared from control cells vs. cells exposed to either titanium dioxide nanoparticles or to copper oxide nanoparticles. The results are detailed in Fig 3, in Table 3, in S2 Fig and S3 Fig.

**Table 3:** Differentially-expressed proteins identified in the proteomic screen.

Spot	Protein name	Access	Nb.	Sequence	TiO2/ctrl	CuO/ctrl	Cu ion/ctrl
Nb.		Number	peptide	Cov. in %	(fold/Ttest)	(fold/Ttest)	(fold/Ttest)
P:	Protein production and folding						
P1	eIF3j	Q66JS6	2	10	0.84/0.81	0.46/0.025	0.79/0.64
P2	Elongation factor 1-delta	P57776	2	13	1.04/0.91	0.7/0.04	1.05/0.34
M:	Metabolism						
M1	Aldose reductase	P45376	4	13	0.995/0.96	0.53/0.02	0.92/0.52
M2	Glutamate DH 1 mitochondrial	P26443	2	4	0.71/0.04	0.98/0.88	0.86/0.27
M3	DLAT, mitochondrial	Q8BMF4	2	6	1.40/0.05	1.12/0.25	0.9/0.16
M4	Galactokinase	Q9R0N0	3	8	1.005/0.97	0.32/0.001	1.005/0.97
S:	Signalling						
S1	Inositol monophosphatase 1	O55023	4	17	0.98/0.96	0.65/0.045	0.71/0.38
S2	Tumor protein D52	Q62393	3	17	0/97/0.68	0.38/0.001	0.83/0.07
S3	Guanine nucleotide-binding protein G	P08752	4	13	0.97/0.85	0.6/0.03	1.02/0.92
O:	Oxidative stress response						
O1	Ferritin light chain 1	P29391	3	19	1.013/0.91	1.65/0.03	1.06/0.75
O2	Ferritin heavy chain	P09528	3	19	2.95/0.35	1.76/0.35	
O3	GSTomega-1	O09131	4	18	1.35/0.15	0.53/0.01	1.23/0.2
O4	Peroxiredoxin-6	O08709	6	33	1.17/0.48	0.83/0.01	1.2/0.23
O5	Glutamate—cysteine ligase reg. subunit	O09172	3	14	0.95/0.83	1.735/0.02	1.22/0.24
O6	Heme oxygenase 1	P14901	3	17	1.02/0.95	6.37/0.05	0.82/0.54
D:	Protein degradation						
D1	Proteasome subunit beta type-9	P28076	2	10	0.66/0.39	0.45/0.01	1.02/0.91
D2	Ubiquitin C-terminal hydrolase L3	Q9JKB1	2	10	1.06/0.88	0.42/0.02	0.65/0.18
C:	Cytoskeleton and trafic						
C1a	MAPREB1 (acidic spot)	Q61166	3	17	a:0.78/0.08	a:0.3/0.001	a: 1.09/0.39
C1b	MAPREB1 (basic spot)	Q61166	4	16	b: 0.92/0.6	b:0.7/0.04	b: 1.03/0.7
C2	Charged MVB protein 4b	Q9D8B3	4	17	1.04/0.85	0.7/0.04	0.85/0.5
C3	Tropomodulin-3	Q9JHJ0	3	10	0.75/0.12	0.54/0.025	1.23/0.03
C4	Stathmin	P54227	6	39	0.83/0.68	0.49/0.04	0.66/0.27
C5	ARP 2/3 complex subunit 5	Q9CPW4	4	27	0.83/0.77	0.22/0.001	0.88/0.1
U:	Miscellaneous						
U1	Apoptosis-associated speck-like protein containing a CARD	Q9EPB4	3	22	1.005/0.95	0.51/0.04	0.91/0.52
U2	Sepiapterin reductase	Q64105	3	15	1.08/0.89	0.76/0.04	1.05/0.91
U3	Tumor protein D52	Q62393	3	17	0/97/0.68	0.38/0.001	0.83/0.07
U4	ATPase Asna1	O54984	3	11	1.58/0.04	0.68/0.123	0.99/0.96
U5	Proliferating cell nuclear antigen	P17918	8	33	0.66/0.15	1.67/0.045	1.12/0.22

With 2180 reproducibly detected spots, and using an average of 3 spots per protein [60], this proteomic screen probed the proteome to a depth of ca 700 gene products, i.e. slightly higher than 10% of the total proteome. Despite this rather limited depth, we could detect reproducible modulations for proteins belonging to several functional classes. The median coefficient of variations of spots was in a range of 10 to 18% depending on the biological conditions, i.e. within the range of typical 2D DIGE experiments, where coefficient of variations range from 18 to 28%, depending on the sample [61, 62]. The protein changes were detected through the use of a variance-based screen [63]. Compared to a fold-change screen, this process compensates automatically for the variance of each spot. This excludes automatically spots with a high coefficient of variation, but enables to take into account small but reproducible changes when the coefficient of variation is low, thus avoiding the arbitrary exclusion of small changes that can be biologically meaningful [63]. In order to take into account the multiple testing issue, the Storey-Tibshirani approach was used [64], and the metrics are shown on S4 Fig. Only the CuO nanoparticles induced significant changes compared to the control cells.

Indeed, several proteins were modulated by the treatment with copper oxide nanoparticles (Table 3), but none of these proteins was modulated when the cells were treated with an equivalent concentration of copper ion, i.e. 125 μM (Table 3), which is in line with the difference of toxicity of nanoparticles compared to the ion. Oppositely, titanium dioxide nanoparticles induced a very limited modulation of protein expression, which is consistent with the recently-published transcriptomic analyses on similar cell types [19]. This also allowed us to use titanium dioxide nanoparticles as a negative control under our conditions of exposure. As both nanoparticles are internalized (Fig 1D and 1E) this shows that the protein modulations observed upon treatment with copper oxide nanoparticles shall not be attributed to the phagocytosis event per se, but to specific effects induced by copper oxide nanoparticles.

### Characterization of the functional responses

As phagocytosis and cytokine production are part of the key functions of macrophages, we tested these functions after treatment with copper oxide and titanium dioxide nanoparticles. In this trend, previous work on cytokine liberation [12] demonstrated that no cytokines were produced upon stimulation by titanium dioxide nanoparticles, as long as the dose remains non toxic.

**Fig 4:**
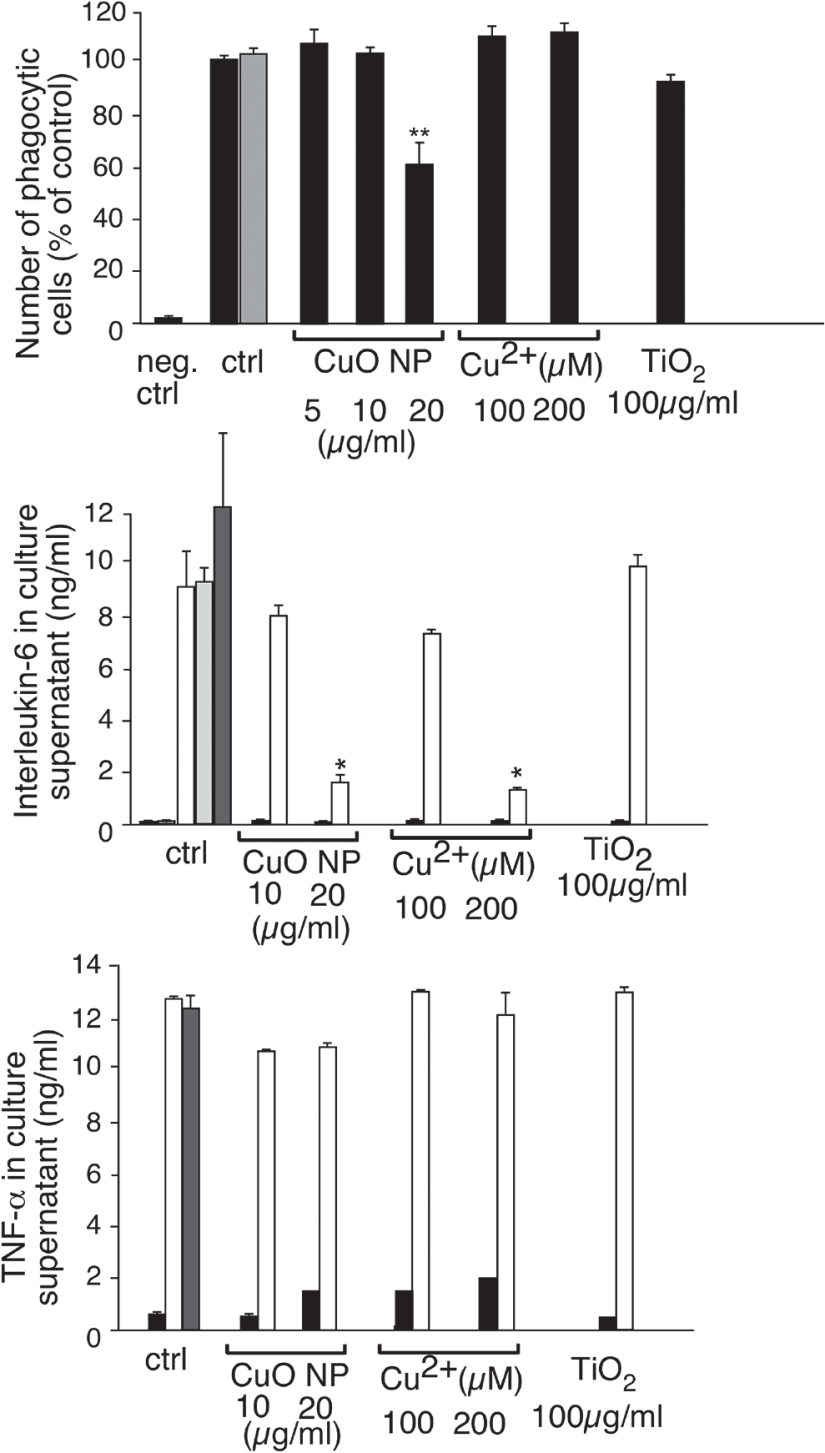
Phagocytic activity and cytokine production of J774 cells upon treatment with particles or copper ion. The phagocytic activity index of the cells is indicated. The results are expressed in percentage of the activity of control cells at 37°C. Negative control: proportion of fluorescent cells when the experiment is carried out without fluorescent beads. Measurements were carried out in triplicate. Statistical confidence (Student T-test) is indicated as follows: *: p ≤ 0.05; ** p ≤ 0.01; *** p≤ 0.001. Grey bar: phagocytic index of cells treated with PVP only (4μg/ml). In panels B and C, the production of respectively IL6 and TNF alpha was assessed. Cells were pretreated for 8 hours with copper ion or with the indicated particles at the indicated concentrations. LPS was then added or not (1μg/ml) and the cells left for an additional 18 hours prior to supernatant harvesting and cytokine measurement. Black bars: cytokine production without LPS stimulation. White bars: cytokine production with LPS stimulation. Grey bars: cytokine production with LPS stimulation and treatment with PVP only (2μg/ml light grey, 4μg/ml dark grey). Hatched bar: cytokine production upon treatment with PVP only (4μg/ml) and without LPS stimulation.

We thus investigated first the phagocytic capacity of the cells and their production of the pro-inflammatory cytokines IL-6 and TNF alpha. The results, shown on Fig 4, demonstrate that the phagocytic capacity of the cells was not altered at non toxic doses of titanium dioxide and copper oxide nanoparticles. As to pro-inflammatory cytokine production, TNF alpha was weakly induced by copper ion and copper oxide nanoparticles, although to much lower levels than those induced by LPS. Upon co-stimulation with LPS, the nanoparticles and the ions did not induced any important change in TNF production. IL-6 showed a very different profile, with no production in the absence of LPS. Titanium dioxide nanoparticles did not significantly modulate the LPS-induced production of IL-6. However, copper ion and copper oxide nanoparticles induced a dose-dependent decrease in the LPS-induced IL-6 production. In all cases, PVP did not show any effect.

Nevertheless, titanium dioxide-treated cells could be functional but altered in some other characteristics, e.g. proliferation. This hypothesis was tested by cell sorting after treatment of the cells with titanium dioxide [65]. The results, displayed in S5 Fig, showed a slightly faster proliferation of titanium dioxide-loaded cells. As the sorting process stresses the cells, it is not possible to determine whether the higher proliferation speed observed is just due to a better resistance of the cells having ingested the particles or is an intrinsic feature of the titanium dioxide-loaded cells.

#### Characterization of the oxidative stress response to copper oxide nanoparticles

As to the peroxiredoxin family, it is noteworthy that only PRX6 is modulated in our experiments, while PRX2 and PRX4 are also very easily detected in our proteomic screen (e.g. in [32, 66]). This is however in line with the general observations on mouse cells [67], especially when a known oxidative stress is involved [68].

In this panel of oxidative stress response proteins, the regulatory subunit of the glutamate cysteine ligase (GCLM protein) deserved some attention, as this protein regulates the synthesis of glutathione [69]. This is of interest in the case of a treatment with copper, as glutathione is part of the copper buffering system in cells [70], the other part being represented by metallothioneins, which are upregulated in a zinc stress [19], but also by copper nanoparticles in hepatoma cells [71].

**Fig 5:**
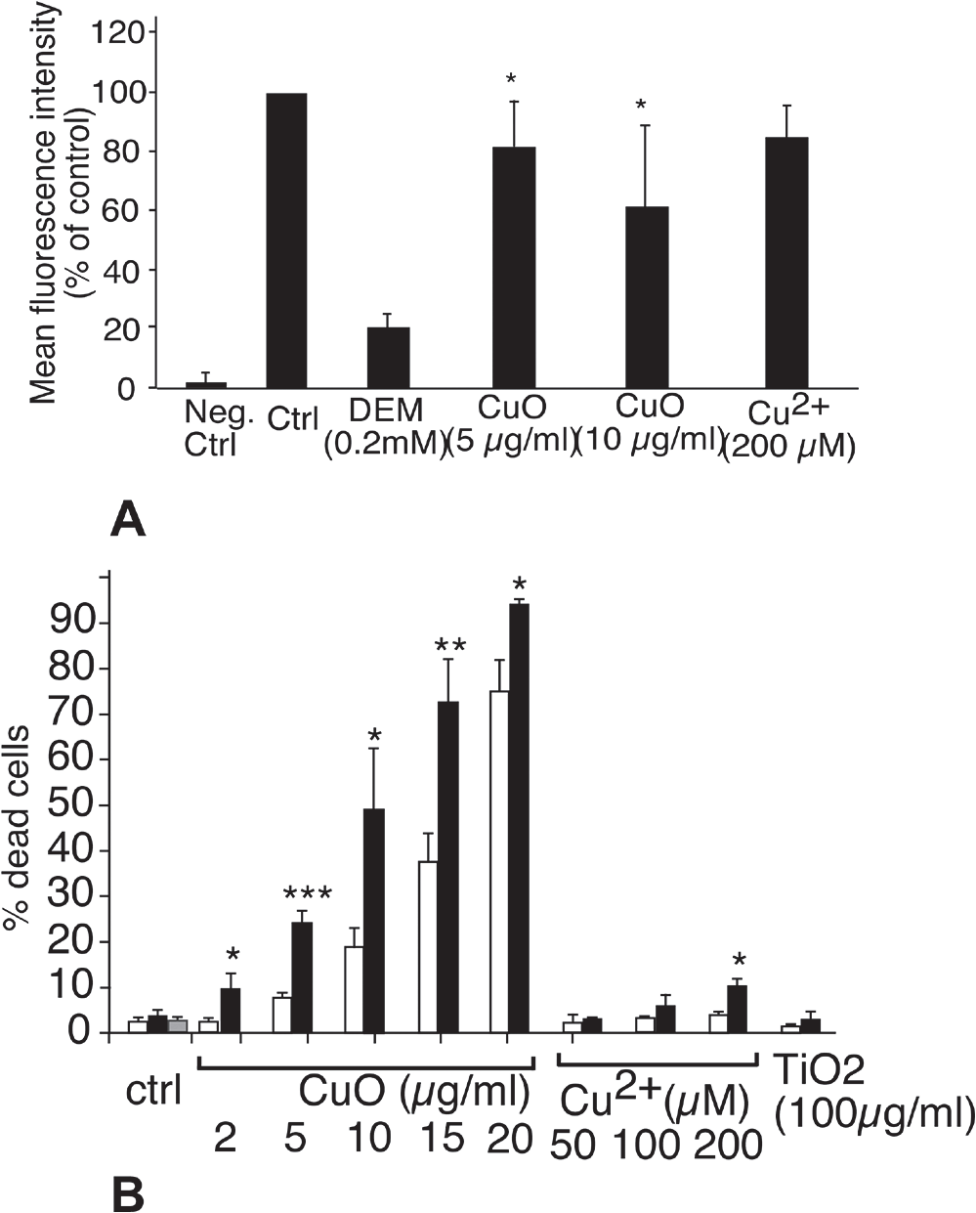
Importance of glutathione in cell survival after treatment with copper. In panel A, the intracellular free glutathione concentration was measured by the monochlorobimane conjugation assay after a 24 hour treatment with copper oxide nanoparticles or copper ion or diethylmaleate (DEM). The results are expressed in % of positive cells compared to the control population. DEM adds to the SH group of glutathione and is used as a control for GSH depletion. In panel B, J774 cells were first treated with 100μM buthionine sulfoximine for 6 hours (black bars) or with vehicle (white bars) Copper oxide nanoparticles or copper ion or titanium dioxide nanoparticles were then added at the indicated concentrations for the remaining 18 hours prior to viability measurement. Grey bar: cells treated with buthionine sulfoximine and PVP (4μg/ml). Experiments were carried out in quadruplicate. Statistical confidence (Student T-test) is indicated as follows: *: p ≤ 0.05; ** p ≤ 0.01; *** p≤ 0.001.

Thus, if the homeostatic hypothesis is verified, the induction of GCLM should represent a mechanism by which cells increase their concentration of glutathione to buffer the copper that has entered the cytoplasm. It is then necessary to determine whether this response is critical for cell survival or whether it belongs to the fitness response. To this purpose, we inhibited the glutamate cysteine ligase with buthionine sulfoximine and checked the effect of this inhibition on cell survival upon treatment with copper. The results, shown in Fig 5, demonstrate that inhibition of glutamate cysteine ligase dramatically increases the sensitivity of the cells to copper oxide nanoparticles, but not to copper ion, except at high concentrations. When combined with the observed data for dissolved copper concentrations for both copper oxide nanoparticles and copper ions, this suggests that the intracellular copper concentration is not the primary determinant of this response. The way and the location at which copper enters into the cells and is present locally within the cell seem to play a critical role.

**Fig 6:**
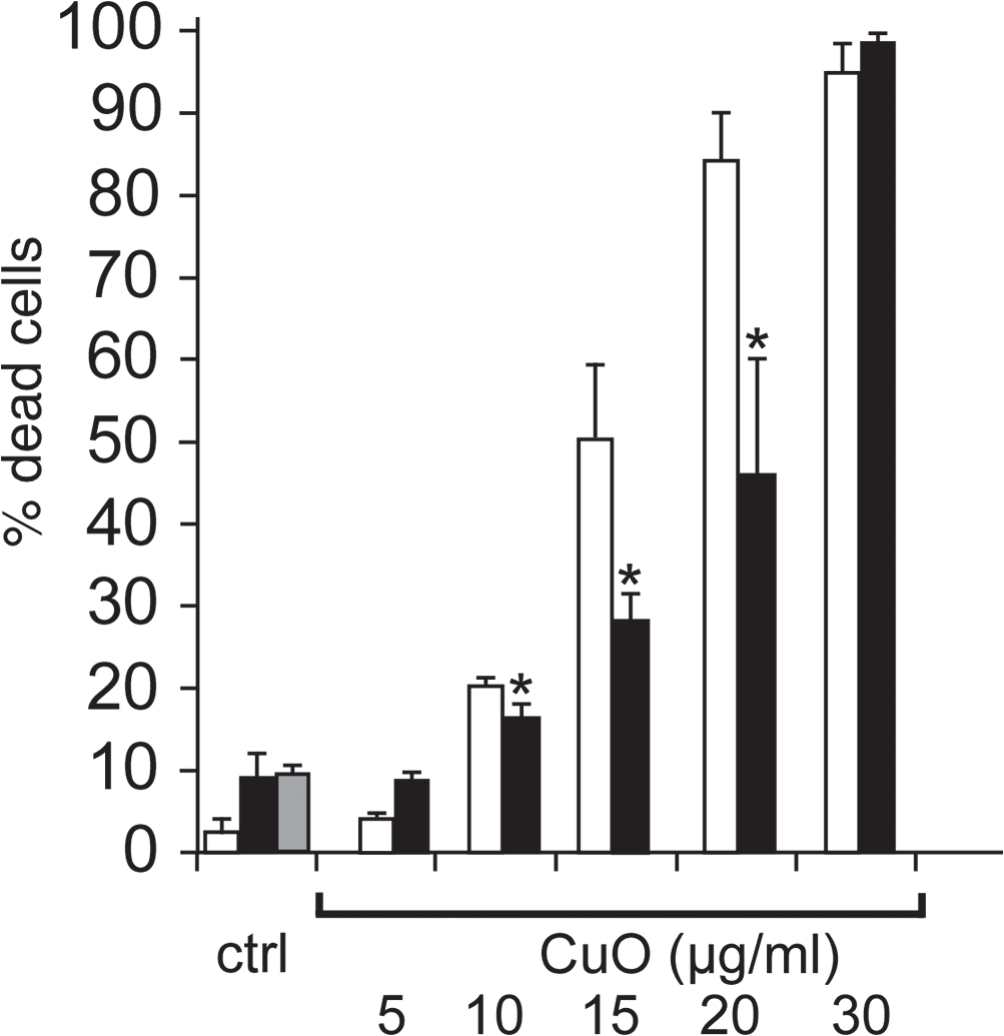
Importance of heme oxygenase induction in cell survival after treatment with copper. J774 cells were first treated with 2μM lovastatin for 6 hours (black bars) or with vehicle (white bars). Copper oxide nanoparticles were then added at the indicated concentrations for the remaining 18 hours prior to viability measurement. Experiments were carried out in quadruplicate. Statistical confidence (Student T-test) is indicated as follows: *: p ≤ 0.05; ** p ≤ 0.01; *** p≤ 0.001.

However, the strongest induction observed was the one of heme oxygenase. Heme oxygenase acts as an antioxidative protein, by cleaving the free heme, which is a strong pro-oxidant, and liberating biliverdin (an antioxidant), CO and free iron. Thus, the strong induction of heme oxygenase could be a protective mechanism of the cells against copper oxide nanoparticles. To test this hypothesis, we pre-induced heme oxygenase overexpression by statins [72] prior to treatment with nanoparticles. The results, displayed on Fig 6, show that heme oxygenase provides a protective mechanism against copper oxide toxicity.

#### The interplay between catechols and copper: a new toxic mechanism for macrophages

**Fig 7:**
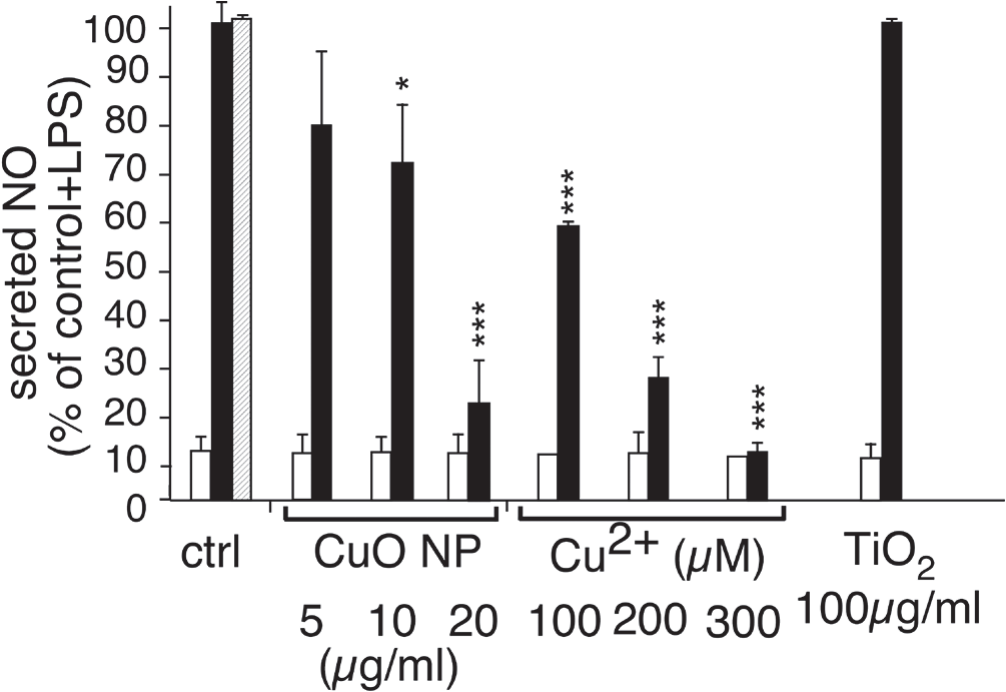
Alteration of NO production. The NO production was evaluated after treatment with particles or copper ion. Black bars, after further stimulation with 1μg/ml of lipopolysaccharide (LPS). White bars, without stimulation with LPS. Hatched bar: stimulation with PVP (4 μg/ml) and LPS. The results are expressed in percentage of the activity of control cells after LPS stimulation. Both measurements were carried out in triplicate on independent cultures and the statistical confidence in the Student T-test is indicated as follows: *: p ≤ 0.05; ** p ≤ 0.01; *** p≤ 0.001.

The purpose of carrying out an omic approach is to evidence physiological mechanisms that are unsuspected from the data available from more classical, targeted approach. In our proteomic screen, sepiapterin reductase was one of the major outliers, standing out of the classical pathways either observed for every type of stress [73], or linked to the classical oxidative stress paradigm. Sepiapterin reductase is the last enzyme in the pathway leading to the synthesis of tetrahydrobiopterin. This cofactor is used in only a few hydroxylation reactions, namely the hydroxylations of phenylalanine (producing tyrosine), of tyrosine (producing DOPA) and of tryptophan (producing 6-hydroxytryptophan). It is also a cofactor of nitric oxide synthases, where it is used for the hydroxylation of arginine in the sequence leading to NO production. On this basis, and keeping in mind the interplay between NO production and glutathione levels [74] and heme oxygenase levels [75], we first investigated the NO production in cells treated with copper oxide nanoparticles or copper ion, either alone or concomitantly with LPS induction. The results (Fig 7) show first that none of the particles tested induces NO production by themselves, and second that only copper ion is able to decrease LPS-induced NO production at non toxic concentrations, a well known observation [76]. The absence of effects of copper oxide nanoparticles is however in line with our proteomic observations, as the induction of GCLM and heme oxygenase have opposite effects in LPS-induced NO production [74]. Moreover, it is highly unlikely that the moderate decrease that we observe in sepiapterin reductase will have any direct effect on the NOS, as an extremely strong depletion in tetrahydrobiopterin is necessary to decrease NO production [77].

**Fig 8:**
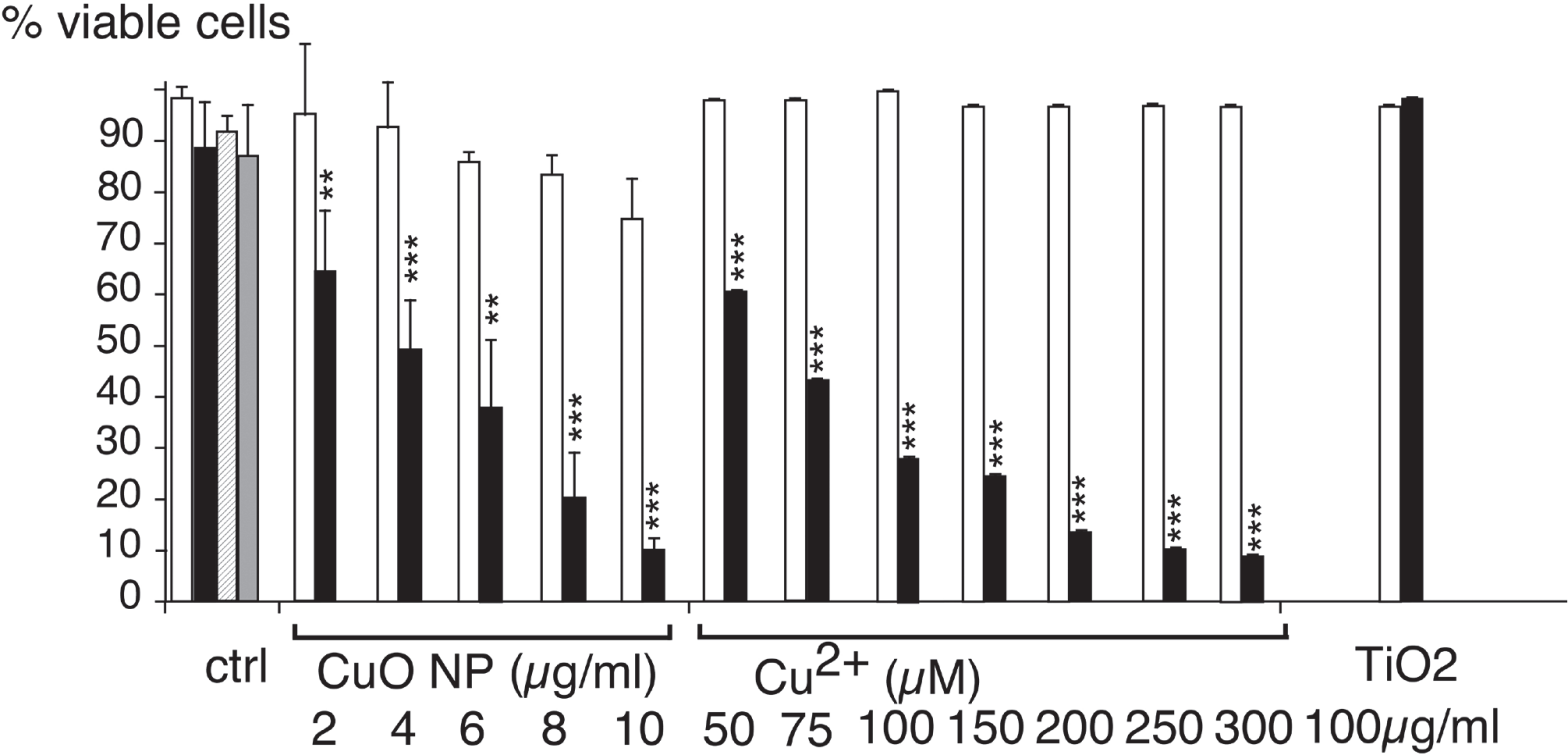
Interplay between DOPA and copper in cell survival. J774 cells were first treated with 100μM DOPA for 6 hours (black bars) or with vehicle (white bars). Copper oxide nanoparticles or copper ion or titanium dioxide nanoparticles were then added at the indicated concentrations for the remaining 18 hours prior to viability measurement. Hatched bar: cells treated with 100μM oxidized DOPA. Grey bar cells treated with PVP only (4μg/ml). Experiments were carried out in quadruplicate. Statistical confidence (Student T-test) is indicated as follows: *: p ≤ 0.05; ** p ≤ 0.01; *** p≤ 0.0011.

Next, we tested whether the tetrahydrobiopterin-dependent amino acid hydroxylations could show an interplay with copper toxicity. As tyrosine has been shown to play a role in zinc resistance [78], the products of the amino acid hydroxylations, i.e. tyrosine, DOPA and 6-hydroxytryptophan were tested by adding them at non toxic doses together with varying copper concentrations. Neither tyrosine nor 6-hydroxytryptophan induced any copper-dependent change in cell survival (data not shown). As shown on Fig 8, however, DOPA induced a strong increase in copper sensitivity, and the contrast is even stronger for copper ion than for copper oxide nanoparticles. To date, such a catechol-dependent copper toxicity has been described only for catecholinergic neurons (e.g. in [79–81]).

## Discussion

In the core of this study, the effects of copper oxide nanoparticles and of titanium dioxide nanoparticles were compared. The rationale was to compare a nanoparticle inducing very limited cellular responses in macrophages, i.e. titanium dioxide [17, 19, 20], to a nanoparticle known to be highly toxic, i.e. copper oxide [26, 33]. Thus, common phenomena, e.g. those strictly linked to the phagocytosis of nanoparticles per se, should induce common responses, while copper oxide nanoparticles-specific responses should be seen differentially. In order to get a broader view of the cellular responses, we used a proteomic screen. This scheme allowed us to highlight pathways modulated by macrophages in response to copper oxide nanoparticles, and then to check the importance of these pathways in cell survival.

Apart from cytoskeleton, which belongs to the dejàvu in proteomics [67], and which has been recently shown to be altered upon treatment of cells with copper oxide nanoparticles [33], the dominating category in our study was the oxidative stress response, which is in line with the well known Fenton reaction catalyzed by copper [82]. We also observed a strong induction of heme oxygenase, confirming previous results obtained with copper [83, 84] and other metals [85]. We could also demonstrate that heme oxygenase induction (e.g. with the widely used statins drugs) had a protective effect.

More surprising is the induction of ferritin a specific iron chelator, by the copper oxide nanoparticles. This could be due to the activity of heme oxygenase, liberating free iron in the cytosol, and needing increased levels of ferritin to complex it and avoid the toxic effects that have been already documented [86], or to a possible direct effect of copper on iron-sulfur proteins, also liberating free iron [87].

Of high interest was the induction of GCLM, the limiting enzyme of glutathione biosynthesis, a phenomenon already observed with zinc [88]. However, and opposite to what was observed with zinc, the free glutathione levels were decreased with copper oxide nanoparticles, suggesting a consumption of glutathione to complex intracellular copper [70]. This critical role of glutathione in resistance to copper was further evidenced by the deleterious effects of the inhibition of de novo glutathione biosynthesis in the presence of copper oxide nanoparticles (Fig 5).

Beside glutathione, the major natural copper chelator in cells is represented by metallothioneins, which are induced by copper [84, 89] and by copper oxide nanoparticles [71]. However, metallothioneins are very difficult to detect in proteomic screens of any type, because of their small size, basic pI and very peculiar amino acid composition. It is therefore not surprising that we did not detect metallothioneins in our proteomic screen.

Apart from these rather classical cellular responses, the most intriguing response seen in our proteomic screen was the modulation of sepiapterin reductase, leading us to the synergy between copper and catechols in terms of toxicity, a phenomenon previously described only for catecholinergic neurons [79–81].

This combined toxic mechanism appears quite complex. On the one hand the catechols increase copper import [80], and on the other hand the oxidation products of catechols exhibit an intrinsic toxicity [79, 80, 90]. It is however highly likely that this toxic mechanism can be transposed to macrophages, as they are able to produce and secrete catecholamines [91, 92]. Functionally speaking, catecholamines modulate the macrophage responses [92–95], as well as their survival by enhancing the oxidative burst. This oxidative burst can increase macrophage survival when fighting an infection [96], while inducing apoptosis when no bacteria are present [91]. Indeed the catecholamine production by macrophages is modulated by external stimuli such as bacterial products [92]. Thus, our results point at potentially dangerous interference of copper with macrophages in physiological conditions, i.e. where bacterial challenges and thus catecholamine production occur frequently.

Last but not least, the comparison between our study and another recent proteomic study on responses of epithelial cells to copper oxide nanoparticles [33] clearly shows that the responses are extremely cell-type dependent. Wide differences have also been observed at the transcriptomic level when the responses of various immune cells to zinc oxide have been investigated [19].

## Conclusions

The multi-faceted comparison of the cellular responses to copper oxide nanoparticles, titanium dioxide nanoparticles and copper ion allowed us to get better insights into the responses of the macrophages to these compounds. In our screen, titanium dioxide nanoparticles did not induce any detectable perturbation of the cellular functions and survival. Conversely, copper ion and copper oxide nanoparticles induced important interferences with some of the essential macrophage functions, e.g. the LPS-induced cytokines or NO production. In addition, our proteomic analyses were able to detect critical responses of macrophages for their survival to copper oxide nanoparticles. At the same time, macrophages treated with copper oxide nanoparticles induced the protective heme oxygenase, ferritin to chelate the iron that may be liberated, increased the glutathione production to chelate copper and decrease their production of catecholamines that become toxic in the presence of copper. This latter interference, documented up to now in catecholinergic neurons only, may be of importance in complex situations where cells are submitted simultaneously to copper and to situations stimulating catecholamines production, e.g. a bacterial challenge. In the case of macrophages, such situations include bacterial challenges or physiological stress, i.e. situations that are very likely to occur under real life conditions

## Supporting Information

S1 Fig TEM images of the nanoparticles used in this study after dispersion. (PDF)

S2 Fig Detail of the 2D gels with highlithed spots part 1. (PDF)

S3 Fig Detail of the 2D gels with highlithed spots part 2. (PDF)

S4 Fig Statistical evaluation of the proteomic screen. (PDF)

S5 Fig Growth ability of titanium dioxide-treated J774 cells. (PDF)

S1 Methods Detailed supplementary material and methods. (PDF)
