# Supplementary material for: Comparative Proteomic Analysis of the Molecular Responses of Mouse Macrophages to Titanium Dioxide and Copper Oxide Nanoparticles Unravels Some Toxic Mechanisms for Copper Oxide Nanoparticles in Macrophages
Source: PLoS One. 2015 Apr 22;10(4):e0124496. doi: 10.1371/journal.pone.0124496 (PMC4406518; doi:10.1371/journal.pone.0124496)
Supplement: S1 Fig — (PDF) [file pone.0124496.s001.pdf]

**Supporting information Figure S1**: TEM images of the nanoparticles used in this study after dispersion.

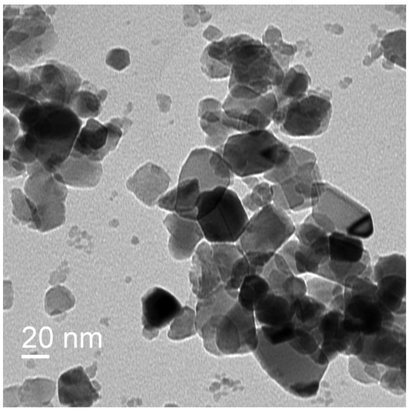


A

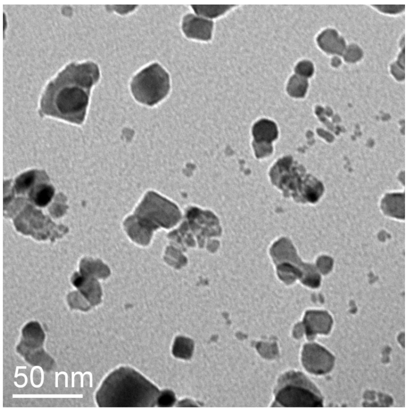


B

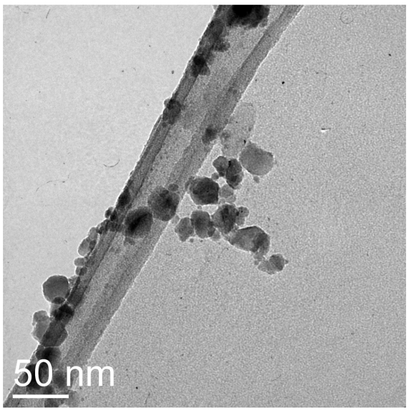


C

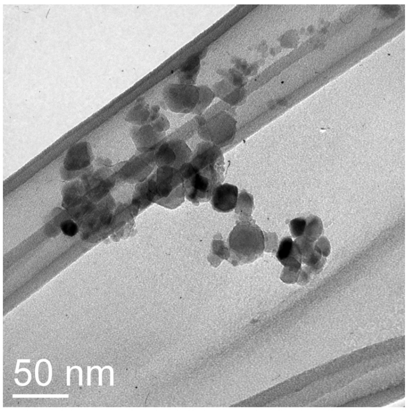


D

A and B: Titanium dioxide

C and D: Copper oxide
