# Supplementary material for: Comparative Proteomic Analysis of the Molecular Responses of Mouse Macrophages to Titanium Dioxide and Copper Oxide Nanoparticles Unravels Some Toxic Mechanisms for Copper Oxide Nanoparticles in Macrophages
Source: PLoS One. 2015 Apr 22;10(4):e0124496. doi: 10.1371/journal.pone.0124496 (PMC4406518; doi:10.1371/journal.pone.0124496)
Supplement: S3 Fig — (PDF) [file pone.0124496.s003.pdf]

Supporting Information Figure S3 : detail of the 2D gels with highlithed spots part 2

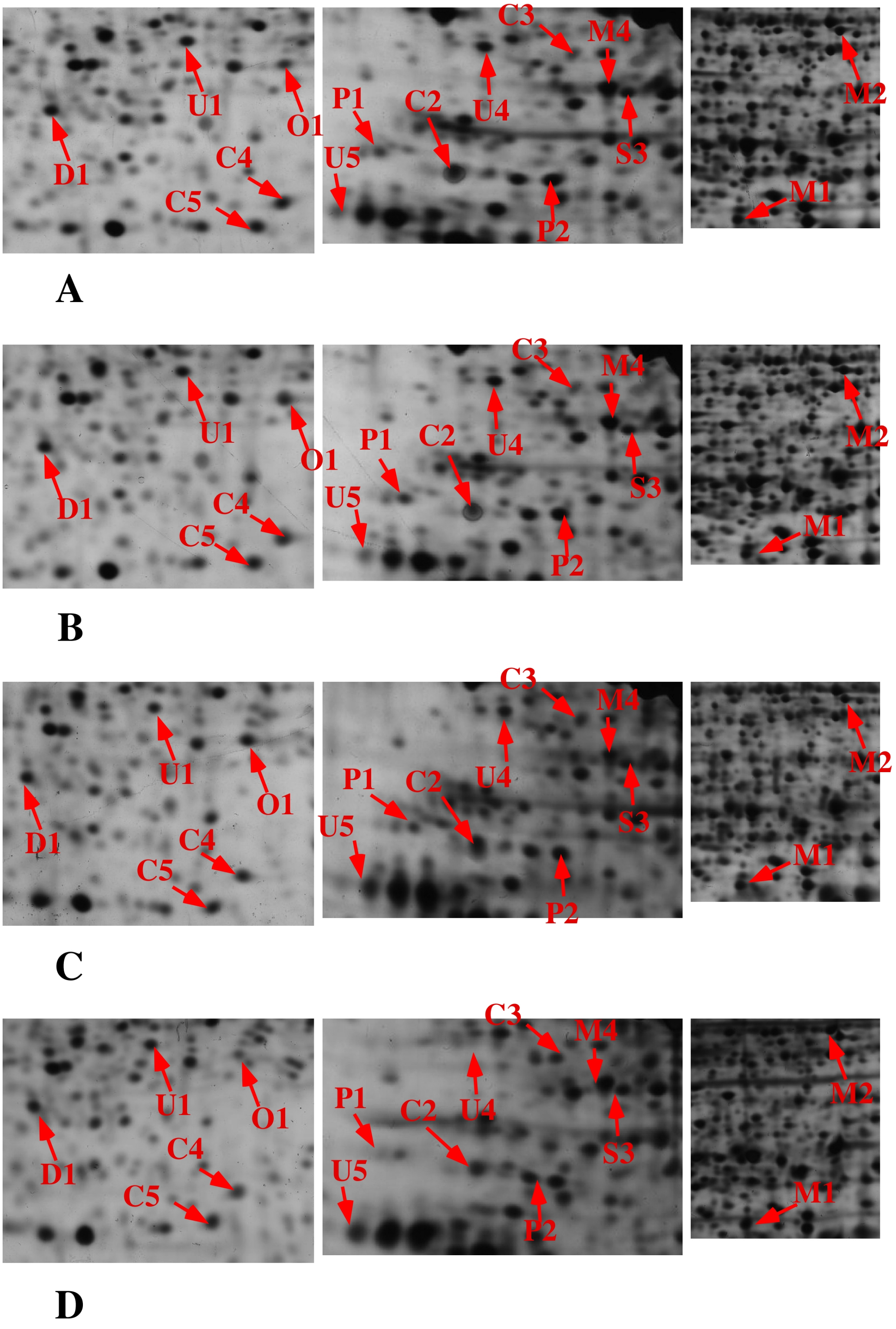

A: gel obtained from control cells
B: gel obtained from cells treated with titanium oxide (100μg/ml, 24 hours)
C: gel obtained from cells treated with copper oxide (10μg/ml, 24 hours)
D: gel obtained from cells treated with copper ions (125μM, 24 hours)

The arrows point to spots that show reproducible and statistically significant changes between the control and NP-treated cells ($p \leq 0.05$). Spot numbering according to Table 3.
