# Supplementary material for: Comparative Proteomic Analysis of the Molecular Responses of Mouse Macrophages to Titanium Dioxide and Copper Oxide Nanoparticles Unravels Some Toxic Mechanisms for Copper Oxide Nanoparticles in Macrophages
Source: PLoS One. 2015 Apr 22;10(4):e0124496. doi: 10.1371/journal.pone.0124496 (PMC4406518; doi:10.1371/journal.pone.0124496)
Supplement: S4 Fig — (PDF) [file pone.0124496.s004.pdf]

Supporting information Figure S4: Statistical evaluation of the proteomic screen

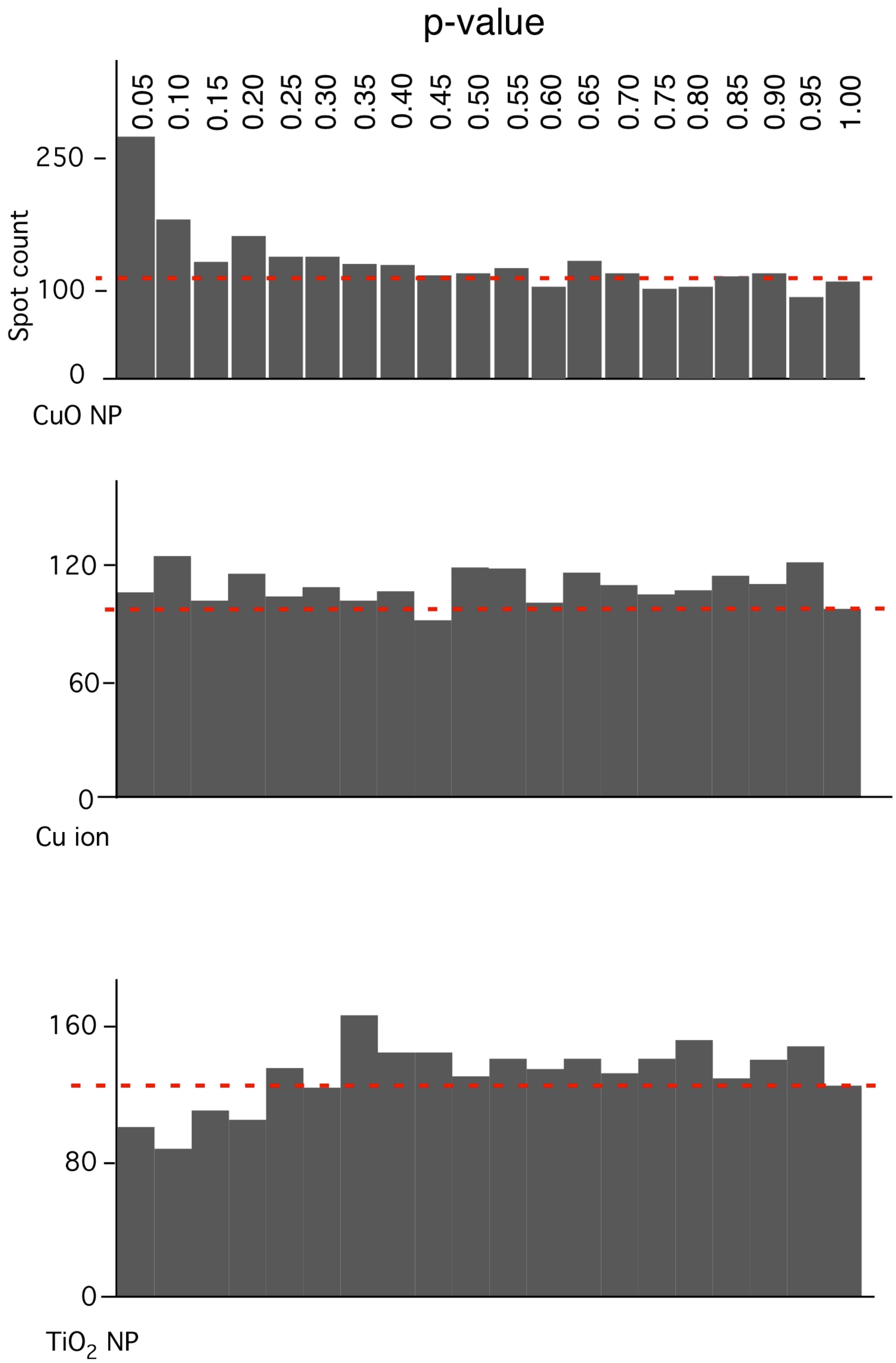

This figure shows the distribution of the t-tests for all spots detected in the image analysis of the 2D gels (4 independent biological replicates per condition). This allows to estimate the proportion of false positives, i.e. spots detected only through random processes, in the selected spots, i.e. those with a t-test lower than 0.05. The dotted line indicates the false positive threshold
