# Supplementary material for: Comparative Proteomic Analysis of the Molecular Responses of Mouse Macrophages to Titanium Dioxide and Copper Oxide Nanoparticles Unravels Some Toxic Mechanisms for Copper Oxide Nanoparticles in Macrophages
Source: PLoS One. 2015 Apr 22;10(4):e0124496. doi: 10.1371/journal.pone.0124496 (PMC4406518; doi:10.1371/journal.pone.0124496)
Supplement: S5 Fig — (PDF) [file pone.0124496.s005.pdf]

Supporting information Figure S5: Growth ability of titanium dioxide-treated J774 cells

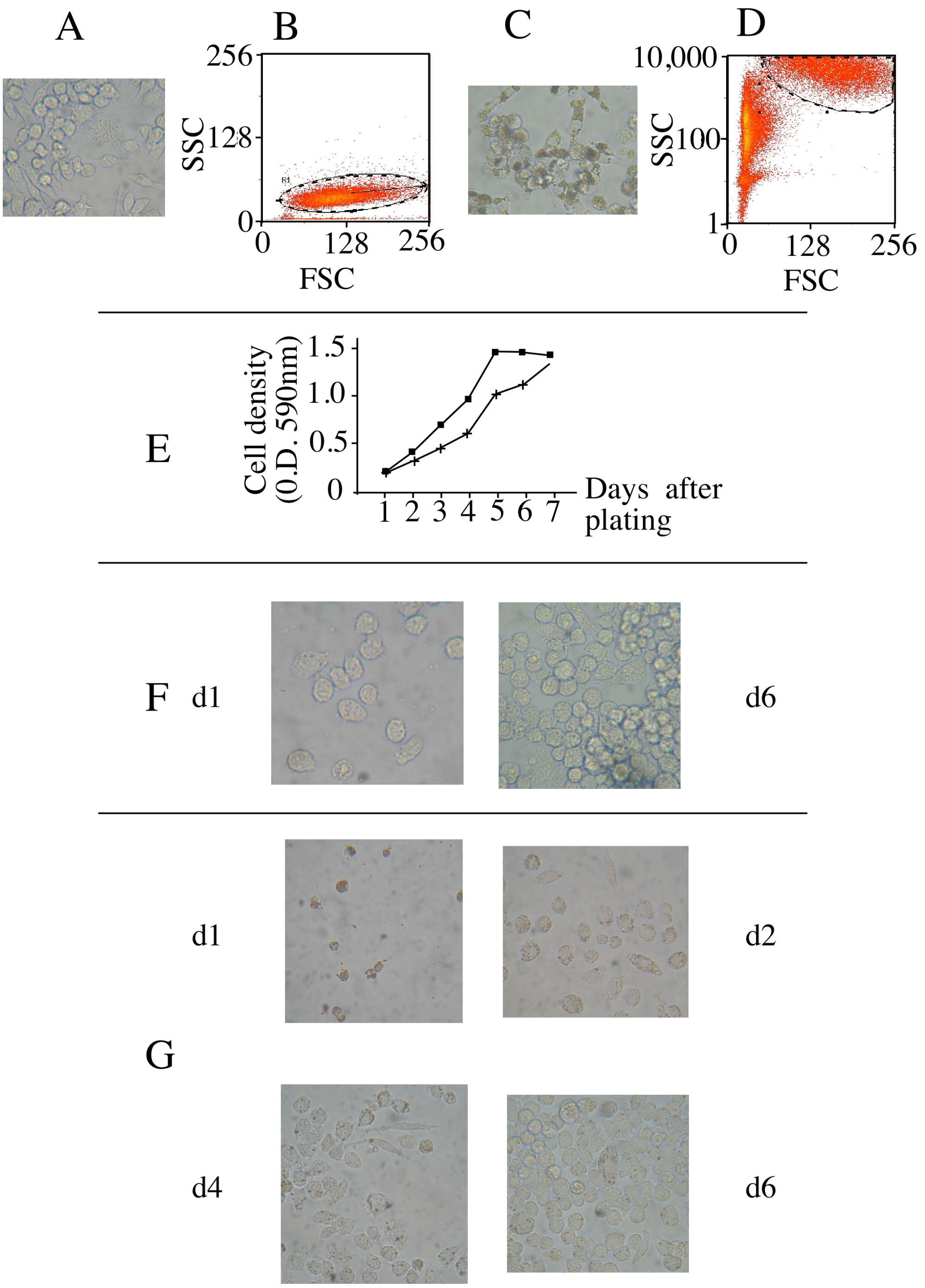

This figure represents the various steps of the sorting and growth experiment.
A : light microscopy image of control cells.
B : scattering parameters of the control cells in the FACS. The dotted line shows the sorting window for control cells
C : light microscopy image of titanium dioxide-treated cells (100μg/ml, 24 hours).
D : scattering parameters of the titanium dioxide-treated cells in the FACS. The dotted line shows the sorting window for titanium dioxide-loaded cells

E : growth curve of the control cells (crosses) and titanium dioxide-treated cells (squares) after sorting and plating at 100,000 cells/ml. Doubling time : 37 hours for control cells and 22 hours for titanium dioxide-treated cells
F : light microscopy image of sorted control cells at day1 and day 6 after plating
G : light microscopy image of sorted, titanium dioxide containing cells at day 1, 2 4 and 6 after plating
