# Supplementary material for: Comparative Proteomic Analysis of the Molecular Responses of Mouse Macrophages to Titanium Dioxide and Copper Oxide Nanoparticles Unravels Some Toxic Mechanisms for Copper Oxide Nanoparticles in Macrophages
Source: PLoS One. 2015 Apr 22;10(4):e0124496. doi: 10.1371/journal.pone.0124496 (PMC4406518; doi:10.1371/journal.pone.0124496)
Supplement: S1 Methods — (PDF) [file pone.0124496.s006.pdf]

1. Supporting Information Material and methods

1.1. Transmission Electron Microscopy for cells

Fixation was initiated by adding an equal volume of fixative solution, previously warmed to 37°C, to the cells after treatment with nanoparticles for 24 hours. The fixative solution contained 5% glutaraldehyde (Electron Microscopy Sciences. Euromedex, Strasbourg, France) in a 0.1 M sodium cacodylate buffer (both Merck, Darmstadt, Germany) (305 mOsm. pH 7.3). After 10 min the mixture was centrifuged, the supernatant was discarded, and the pellet resuspended in the fixative solution containing 2.5% glutaraldehyde in 0.1 M sodium cacodylate buffer for 45 min at room temperature. The cells were then washed in 0.1 M sodium cacodylate buffer, postfixed for 1 h at 4°C with 1% osmium tetroxide (Merck) in the same buffer and stained for 1 h at 4°C in 4% uranyl acetate. After further washing in distilled water, the cells were dehydrated in graded (50, 70, 80, 95, and 100%) ethanol solutions, incubated for 1 h in Epon (Electron Microscopy Sciences):absolute alcohol (1:1. vol/vol), then overnight in Epon and embedded in Epon. Ultrathin sections, stained with lead citrate (*Leica*, Bron, France) and uranyl acetate (Merck), were examined under a Philips CM 120 BioTwin electron microscope (120 kV).

1.2. Intracellular glutathione measurements

Cells were harvested, centrifuged for 5 minutes and then labelled for 5 minutes with a 50 μM monochlorobimane solution diluted in PBS at 37°C. The reaction was stopped for 5 minutes on ice in the dark. After two washes in cold PBS, the cells were analyzed by flow cytometry on a MoFlo instrument (Beckman Coulter) using a UV laser for excitation and reading the emission at 450 nm.

1.3. Phagocytosis activity measurement

The phagocytic activity was measured using fluorescent latex beads (1μm diameter, green labelled, Sigma #L4655) and flow cytometry. Briefly, the beads were pre-incubated at a final concentration of 55 mg/ml for 30 minutes at 37°C in a 1:1 (v/v) PBS/ horse serum solution. They were then incubated with the cells (5.5 mg/L final concentration) for 2 h at 37°C. The cells were then harvested, washed twice with cold

PBS, and incubated for 5 minutes on ice with a 0.25 M D-sorbitol solution in order to remove beads adhering to the cell surface. After another wash with cold PBS, cells were analyzed by flow cytometry on a FACScalibur instrument (Beckton Dickinson).

1.4. Cell sorting

Cells were first incubated for 24 hours with 100μg/ml titanium dioxide. They were then harvested, washed in PBS, and sorted according to their light scattering parameters in a MoFlo cell sorter. Control cells were treated and sorted with exactly the same protocol. The sorted cells were then recovered by centrifugation and seeded in multiwell plates at 100,000 cells/ml in a complete cell culture medium with antibiotics and nystatin (20μg/ml) as a fungicide. Cells were then grown for a week. Every day, wells were submitted to fixation and evaluation of cell numbers by the crystal violet assay.

1.5. Proteomics

1.5.1. 2D gel electrophoresis

isoelectric focusing

Home made 160 mm long 4-8 linear pH gradient gels were cast according to published procedures. Four mm-wide strips were cut, and rehydrated overnight with the sample, diluted in a final volume of 0.6 ml of rehydration solution (7 M urea, 2 M thiourea, 4% CHAPS, 0.4% carrier ampholytes (Pharmalytes 3-10) and 100mM dithiodiethanol.

The strips were then placed in a Multiphor plate (GE Healthcare), and IEF was carried out with the following electrical parameters: 100V for 1 hour, then 300V for 3 hours, then 1000V for 1 hour, then 3400 V up to 60-70 kVh. After IEF, the gels were equilibrated for 20 minutes in Tris 125mM, HCl 100mM, SDS 2.5%, glycerol 30% and urea 6 M . They were then transferred on top of the SDS gels and sealed in place with 1% agarose dissolved in Tris 125mM, HCl 100mM, SDS 0.4% and 0.005% (w/v) bromophenol blue.

SDS electrophoresis and protein detection

Ten percent gels (160x200x1.5 mm) were used for protein separation. The Tris taurine buffer system was used and operated at a ionic strength of 0.1 and a pH of

7.9. The final gel composition is thus Tris 180mM, HCl 100 mM, acrylamide 10% (w/v), bisacrylamide 0.27%. The upper electrode buffer is Tris 50 mM, Taurine 200 mM, SDS 0.1%. The lower electrode buffer is Tris 50 mM, glycine 200 mM, SDS 0.1%. The gels were run at 25V for 1hour, then 12.5W per gel until the dye front has reached the bottom of the gel. Detection was carried out by fast silver staining.

1.5.2. Image analysis

The gels were scanned after silver staining on a flatbed scanner (Epson perfection V750), using a 16 bits grayscale image acquisition. The gel images were then analyzed using the Delta 2D software (v 3.6). Three gels coming from three independent cultures were used for each experimental group. Spots that were never expressed above 100 ppm of the total spots were first filtered out. Then, significantly-varying spots were selected on the basis of their Student T-test p-value between the treated and the control groups. Spots showing a p-value lower than 0.05 were selected.

1.5.3. Mass spectrometry

In gel digestion was performed with an automated protein digestion system, MassPrep Station (Waters, Milford, USA). The gel plugs were washed twice with 50 μL of 25 mM ammonium hydrogen carbonate (NH_4HCO_3) and 50 μL of acetonitrile. The cysteine residues were reduced by 50 μL of 10 mM dithiothreitol at 57°C and alkylated by 50 μL of 55 mM iodoacetamide. After dehydration with acetonitrile, the proteins were cleaved in gel with 10 μL of 12.5 ng/μL of modified porcine trypsin (Promega, Madison, WI, USA) in 25 mM NH_4HCO_3. The digestion was performed overnight at room temperature. The generated peptides were extracted with 30 μL of 60% acetonitrile in 0.1% formic acid. Acetonitrile was evaporated under vacuum before nanoLC-MS/MS analysis.

NanoLC-MS/MS analysis was performed using on nanoLC-QTOF-MS system and on nanoLC-IT-MS system.

The nanoLC-QTOF-MS system was composed of the nanoACQUITY Ultra-Performance-LC (Waters Corporation, Milford, USA) coupled to the Synapt™ High

Definition Mass Spectrometer^{TM} (Waters Corporation, Milford, USA). The system was fully controlled by MassLynx 4.1 SCN639 (Waters Corporation, Milford, USA).

The nanoLC system was composed of ACQUITY UPLC® BEH130 C18 column (250 mm x 75 μm with a 1.7 μm particle size, Waters Corporation, Milford, USA) and a Symmetry C18 precolumn (20 mm × 180 μm with a 5 μm particle size, Waters Corporation, Milford, USA). The solvent system consisted of 0.1% formic acid in water (solvent A) and 0.1% formic acid in acetonitrile (solvent B). 4 μL of sample were loaded into the enrichment column during 3 min at 5 μL/min with 99% of solvent A and 1% of solvent B. Elution of the peptides was performed at a flow rate of 300 nL/min with a 8-35% linear gradient of solvent B in 9 minutes.

The tandem mass spectrometer was equipped with a Z-spray ion source and a lock mass system. The capillary voltage was set at 2.8 kV and the cone voltage at 35 V. Mass calibration of the TOF was achieved using fragment ions from Glu-fibrino-peptide B on the [50;2000] m/z range. Online correction of this calibration was performed with Glu-fibrino-peptide B as the lock-mass. The ion $(M+2H)^{2+}$ at m/z 785.8426 was used to calibrate MS data and the fragment ion $(M+H)^{+}$ at m/z 684.3469 was used to calibrate MS/MS data during the analysis. The system was operated in Data-Dependent-Acquisition (DDA) mode with automatic switching between MS (0.5 s/scan on m/z range [150;1700]) and MS/MS modes (0.5 s/scan on m/z range [50;2000]). The two most abundant peptides (intensity threshold 20 counts/s), preferably doubly and triply charged ions, were selected on each MS spectrum for further isolation and CID fragmentation using collision energy profile. Fragmentation was performed using argon as the collision gas.

Mass data collected during analysis were processed and converted into .pkl files using ProteinLynx Global Server 2.3 (Waters Corporation, Milford, USA). Normal background subtraction type was used for both MS and MS/MS with 5% threshold and polynomial correction of order 5. Smoothing was performed on MS/MS spectra (Savitsky-Golay, 2 iterations, window of 3 channels). Deisotoping was applied for both MS (medium desisotoping) and MS/MS (fast deisotoping).

The nanoLC-IT-MS system was composed of the Agilent 1200 series nanoLC-Chip system (Agilent Technologies, Palo Alto, USA) coupled to the amaZon ion trap

(Bruker Daltonics GmbH, Bremen, Germany). The system was fully controlled by HyStar 3.2 and trapControl 7.0 (Bruker Daltonics, Bremen, Germany).

The chip was composed of a Zorbax 300SB-C18 (43 mm × 75 m, with a 5 m particle size) analytical column and a Zorbax 300SB-C18 (40 nL, 5 m) enrichment column. The solvent system consisted of 2% acetonitrile, 0.1% formic acid in water (solvent A) and 2% water, 0.1% formic acid in acetonitrile (solvent B). 4 μL of sample were loaded into the enrichment column at a flow rate set to 3.75L/min with solvent A. Elution of the peptides was performed at a flow rate of 300 nL/min with a 8-40% linear gradient of solvent B in 7 minutes.

For tandem MS experiments, the system was operated in Data-Dependent-Acquisition (DDA) mode with automatic switching between MS and MS/MS. The voltage applied to the capillary cap was optimized to -1850V. The MS scanning was performed in the standard enhanced resolution mode at a scan rate of 8100 m/z per second. The mass range was 250-1500 m/z. The Ion Charge Control was 200000 and the maximum accumulation time was 200 ms. A total of 2 scans was averaged to obtain a MS spectrum and the rolling average was 1. The six most abundant precursor ions with an isolation width of 4 m/z were selected on each MS spectrum for further isolation and fragmentation. The MS/MS scanning was performed in the ultrascan mode at a scan rate of 32500 m/z per second. The mass range was 100-2000 m/z. The Ion Charge Control was 300000. A total of 2 scans was averaged to obtain an MS/MS spectrum.

Mass data collected during analysis were processed and converted into .mgf files using DataAnalysis 4.0 (Bruker Daltonics GmbH, Bremen, Germany). A maximum number of 1700 compounds was detected with an intensity threshold of 150000. A charge deconvolution was applied on the MS full scan and the MS/MS spectra with an abundance cutoff of 5% and 2% respectively and with a maximum charge state of 3 and 2 respectively.

For protein identification, the MS/MS data were interpreted using a local Mascot server with MASCOT 2.4.0 algorithm (Matrix Science, London, UK) against UniProtKB/SwissProt (version 2012_08, 537,505 sequences). The research was carried out in all species. A maximum of one trypsin missed cleavage was allowed. Spectra from Qtof were searched with a mass tolerance of 15 ppm for MS and 0.05

Da for MS/MS data and spectra from Ion Trap were searched with a mass tolerance of 0.3 Da in MS and MS/MS modes. Carbamidomethylation of cysteine residues and oxidation of methionine residues were specified as variable modifications. Protein identifications were validated with at least two peptides with Mascot ion score above 20.
